# PCR-Dipstick-Oriented Surveillance and Characterization of *mcr-1*- and Carbapenemase-Carrying *Enterobacteriaceae* in a Thai Hospital

**DOI:** 10.3389/fmicb.2019.00149

**Published:** 2019-02-08

**Authors:** Rathina Kumar Shanmugakani, Yukihiro Akeda, Yo Sugawara, Warawut Laolerd, Narong Chaihongsa, Suntariya Sirichot, Norihisa Yamamoto, Hideharu Hagiya, Daiichi Morii, Yoshihiro Fujiya, Isao Nishi, Hisao Yoshida, Dan Takeuchi, Noriko Sakamoto, Kumthorn Malathum, Pitak Santanirand, Kazunori Tomono, Shigeyuki Hamada

**Affiliations:** ^1^Research Institute for Microbial Diseases, Osaka University, Suita, Japan; ^2^Department of Infection Control and Prevention, Graduate School of Medicine, Osaka University, Suita, Japan; ^3^Division of Infection Control and Prevention, Osaka University Hospital, Suita, Japan; ^4^Faculty of Medicine Ramathibodi Hospital, Mahidol University, Bangkok, Thailand; ^5^Laboratory of Clinical Investigation, Osaka University Hospital, Suita, Japan

**Keywords:** *mcr-1*, carbapenemase, *Enterobacteriaceae*, PCR-dipstick, rapid detection

## Abstract

Colistin is used as an alternative therapeutic for carbapenemase-producing *Enterobacteriaceae* (CPE) infections which are spreading at a very high rate due to the transfer of carbapenemase genes through mobile genetic elements. Due to the emergence of *mcr-1*, the plasmid-mediated colistin resistance gene, *mcr-1*-positive *Enterobacteriaceae* (MCRPEn) pose a high risk for the transfer of *mcr-1*-carrying plasmid to CPE, leading to a situation with no treatment alternatives for infections caused by *Enterobacteriaceae* possessing both *mcr-1* and carbapenemase genes. Here, we report the application of PCR-dipstick-oriented surveillance strategy to control MCRPEn and CPE by conducting the PCR-dipstick technique for the detection of MCRPEn and CPE in a tertiary care hospital in Thailand and comparing its efficacy with conventional surveillance method. Our surveillance results showed a high MCRPEn (5.9%) and CPE (8.7%) carriage rate among the 219 rectal swab specimens examined. Three different CPE clones were determined by pulsed-field gel electrophoresis (PFGE) whereas only two MCRPEn isolates were found to be closely related as shown by single nucleotide polymorphism-based phylogenetic analysis. Whole genome sequencing (WGS) and plasmid analysis showed that MCRPEn carried *mcr-1* in two plasmids types—IncX4 and IncI2 with ~99% identity to the previously reported *mcr-1*-carrying plasmids. The identification of both MCRPEn and CPE in the same specimen indicates the plausibility of plasmid-mediated transfer of *mcr-1* genes leading to the emergence of colistin- and carbapenem-resistant *Enterobacteriaceae*. The rapidity (<2 h) and robust sensitivity (100%)/specificity (~99%) of PCR-dipstick show that this specimen-direct screening method could aid in implementing infection control measures at the earliest to control the dissemination of MCRPEn and CPE.

## Introduction

Since carbapenems are regarded as the last line of defense for several multidrug-resistant bacterial infections, carbapenem-resistant organisms impede the effective treatment options and increase the mortality rate of afflicted patients (Kizny Gordon et al., 2017). Amongst different carbapenem-resistant organisms, carbapenemase-producing *Enterobacteriaceae* (CPE) are posing a global health threat due to their hasty dissemination, enabled through the horizontal transfer of carbapenemase genes (van Duin and Doi, 2017). Despite the adverse side-effects, colistin is used as a therapeutic alternative for multidrug-resistant organisms including CPE (Mansour et al., 2017; Poirel et al., 2017). However, due to the high usage of colistin in agriculture and livestock, colistin-resistant organisms have emerged as a serious threat to both the animals and humans (Caniaux et al., 2017; Wang et al., 2017b). Furthermore, clinical isolates of colistin-resistant Gram-negatives are being increasingly reported (Kontopidou et al., 2011; Thi Khanh Nhu et al., 2016). Several *Enterobacteriaceae* species are known to be intrinsically resistant to colistin, however, after the description of *mcr-1*, the plasmid-encoded colistin resistance gene, *mcr-1*-producing *Enterobacteriaceae* (MCRPEn) have been reported worldwide (Olaitan et al., 2014; Liu et al., 2016; Matamoros et al., 2017; Wang et al., 2018). Since multidrug-resistant CPE are well-known for their acquisition of plasmid carrying carbapenemase genes, it is suspected that they could also acquire the *mcr-1*-carrying plasmid from MCRPEn which might lead to the emergence of the superbug resistant to both colistin and carbapenem without alternate treatment regimens. Recent reports also show the sporadic identification of *Enterobacteriaceae* co-harboring carbapenemase and *mcr-1* in plasmids (Beyrouthy et al., 2017; Pulss et al., 2017; Mendes et al., 2018).

Based on the currently available reports, MCRPEn are known for their prevalence in the environment from which they can spread to humans (Guenther et al., 2017; Kieffer et al., 2017). Few studies have reported on the epidemiology of clinical MCRPEn, while other reports screened for *mcr-1* from the previous collections of clinical isolates (Chen et al., 2017; Saly et al., 2017; Terveer et al., 2017; Wang et al., 2017a; Zhong et al., 2018). In Thailand, there have been sporadic cases of *mcr-1*-positive *Escherichia coli* isolated from the clinical specimens of patients (Paveenkittiporn et al., 2017; Srijan et al., 2018). However, there have been no reports on the carriage of MCRPEn in hospitalized patients in the country. Similarly, the epidemiology of CPE carriage in Thai hospitals is still unknown. Thus, there is a lack of epidemiological data on the prevalence of MCRPEn and CPE to initiate efficient infection control measures.

To control their nosocomial transmission/infection, it is necessary to determine the fecal carriage rate of MCRPEn along with CPE in the hospitalized patients. In order to implement infection control measures instantly, a rapid detection system for the simultaneous detection of MCRPEn and CPE would be beneficial. Previously, we established the PCR-dipstick technique for the detection of CPE possessing *bla*_NDM_, *bla*_KPC_, *bla*_IMP_, and *bla*_OXA−48_ carbapenemase genes (Shanmugakani et al., 2017). Then, we included two more carbapenemase genes (*bla*_VIM_, *bla*_GES_) for CPE detection and *mcr-1*, the most prevalent *mcr-1* gene variant for MCRPEn detection (Matamoros et al., 2017; Wang et al., 2018). Thus, the PCR-dipstick could be utilized for the rapid detection of MCRPEn and CPE directly from the clinical specimens by targeting *mcr-1* and six carbapenemase (*bla*_NDM_, *bla*_KPC_, *bla*_IMP_, *bla*_OXA−48_, *bla*_VIM_, and *bla*_GES_) genes, respectively. Here, we attempted to develop a PCR-dipstick-oriented surveillance strategy for determining the MCRPEn and CPE fecal carriage and examined its efficacy by comparing with the conventional method in a tertiary care hospital in Thailand. Next, we analyzed the resistance profile and clonal relatedness of the MCRPEn and CPE isolates. Genetic characterization including multi-locus sequence typing and plasmid analysis of the MCRPEn isolates was carried out by whole genome sequencing (WGS) to determine the characteristics that mediate their mode of transmission. Furthermore, the clinical characteristics of the patients were analyzed to determine the risk factors that influence MCRPEn and CPE carriage in the hospital.

## Results

### Prevalence and Characteristics of MCRPEn and CPE

**Figure 1:**
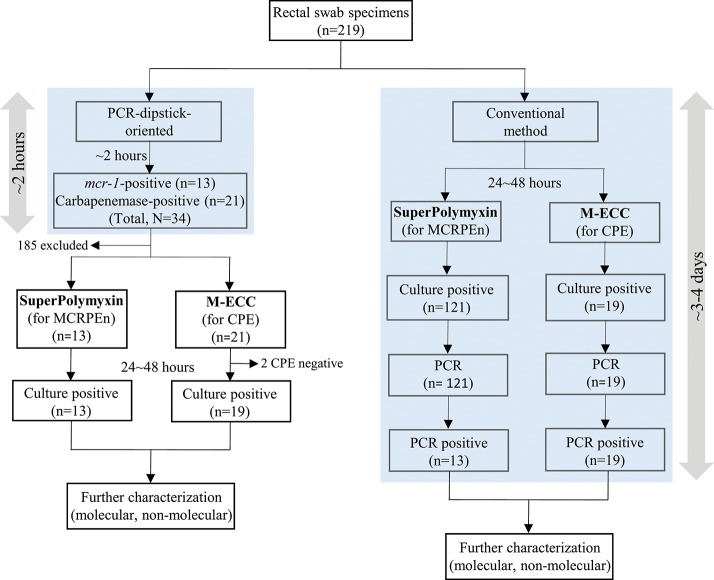
Flow diagram of PCR-dipstick-oriented and conventional surveillance method. All the rectal swab specimens were subjected to PCR-dipstick-oriented and conventional surveillance method. PCR-dipstick detected the presence of resistance genes within 2 h and thus, only the specimens detected with resistance genes were cultured on selective media for the isolation of MCRPEn and CPE. In the conventional method, all of the specimens were cultured on the selective media and all the isolates grown (24~48 h) on them were screened for the resistance genes using PCR.

MCRPEn and CPE surveillance was conducted using both the PCR-dipstick-oriented method and conventional method to examine the efficacy of PCR-dipstick-oriented surveillance strategy (Figure 1). The surveillance results showed that out of the 219 rectal swab specimens collected from the hospitalized patients in a Thai hospital, 13 specimens (5.9%) carried MCRPEn with the *mcr-1* gene and 19 specimens (8.7%) carried CPE with two different carbapenemase genes—*bla*_NDM−1_ (*n* = 7), *bla*_OXA−232_ (*n* = 10), or both (*n* = 2). Two different species of MCRPEn (*E. coli* and *Klebsiella pneumoniae*) and two different species of CPE (*K. pneumoniae* and *Citrobacter farmeri*) were identified (Table 1). Two MCRPEn-positive specimens carried both *E. coli* and *K. pneumoniae* with *mcr-1* gene and thus, we found 15 MCRPEn isolates from 13 MCRPEn-positive specimens. Two specimens were found to carry both MCRPEn and CPE isolates whereas there were no isolates carrying both carbapenemase and *mcr-1* genes. Of the two specimens with both MCRPEn and CPE isolates, one carried *K. pneumoniae* with *bla*_NDM−1_/*bla*_OXA−232_ and *E. coli* with *mcr-1* and the other specimen carried *K. pneumoniae* with *bla*_NDM−1_ and *E. coli* with *mcr-1*. However, neither the MCRPEn were identified with carbapenemase genes nor were the CPE shown to possess the *mcr-1* gene. Antimicrobial susceptibility testing of MCRPEn and CPE showed a varied resistance profile against diverse antibiotics including carbapenems and other β-lactams, fluoroquinolones, tetracyclines, aminoglycosides, sulphonamides, and colistin (Table S1). Notably, all the MCRPEn isolates (15/15) were resistant to colistin with a minimum inhibitory concentration of ≥4 μg/ml.

**Table 1:** Bacterial species and the *mcr-1*/carbapenemsae genes of MCRPEn and CPE isolated from 219 rectal swab specimens.

**Isolate type**	**No. of positive specimens (%)**	**Species (no. of specimens)**	**Resistance genes**	
			**Family**	**Variants**
MCRPEn (*n* = 15)	13 (5.9)	*E. coli* (11)	*mcr-1*	*mcr-1*
		*K. pneumoniae* and *E. coli* (2)	*mcr-1*	*mcr-1*
CPE (*n* = 19)	19 (8.7)	*K. pneumoniae* (6)	*bla*_NDM_	*bla*_NDM−1_
		*C. farmeri* (1)	*bla*_NDM_	*bla*_NDM−1_
		*K. pneumoniae* (10)	*bla*_OXA−48_	*bla*_OXA−232_
		*K. pneumoniae* (2)	*bla*_NDM_/*bla*_OXA−48_	*bla*_NDM−1_/*bla*_OXA−232_

### Clonal Relatedness of MCRPEn and CPE Isolates

**Figure 2:**
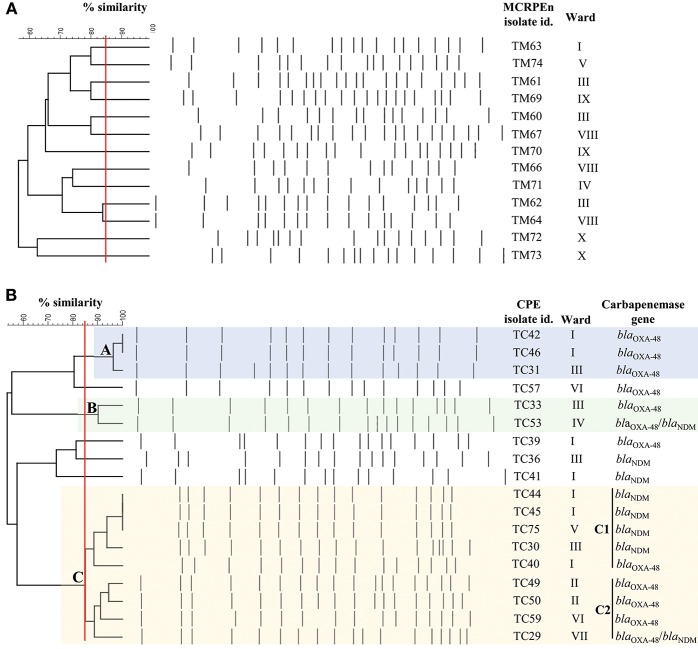
PFGE analysis of *Xba*I-digested MCRPEn (*E. coli*) and CPE (*K. pneumoniae*) isolates. **(A)** Clonal relatedness of 13 *mcr-1*-producing *E. coli* isolates. **(B)** Clonal relatedness of 18 carbapenemase-producing *K. pneumoniae* isolates. A, B, C1, and C2 represents the cluster of isolates with ≥85% similarity (red line). Isolates with Dice similarity coefficient ≥85% fall under one cluster.

Pulsed-field gel electrophoresis (PFGE) was performed after *Xba*I digestion of the 12 MCRPEn (*E. coli*) and 18 CPE (*K. pneumoniae*) isolates to determine their clonal relatedness. It was found that none of the MCRPEn isolates belong to the same clone (≥85% Dice similarity) suggesting that there was no clonal spreading of MCRPEn in the hospital (Figure 2A). Among the 18 *K. pneumoniae* CPE isolates, we found three different clusters, namely A, B, and C (C1 and C2), of isolates with ≥85% Dice similarity (Figure 2B). The isolates in all the three clusters were isolated from the patients admitted not only in the same ward but also in different wards. The isolates in cluster A carry *bla*_OXA−48_ family, in B carry *bla*_OXA−48_ and *bla*_OXA−48_/*bla*_NDM_ family, in C1 carry *bla*_OXA−48_ and *bla*_NDM_ and in C2 carry *bla*_OXA−48_ and *bla*_OXA−48_/*bla*_NDM_ as confirmed by PCR.

### Genomic Characteristics of MCRPEn Isolates

**Figure 3:**
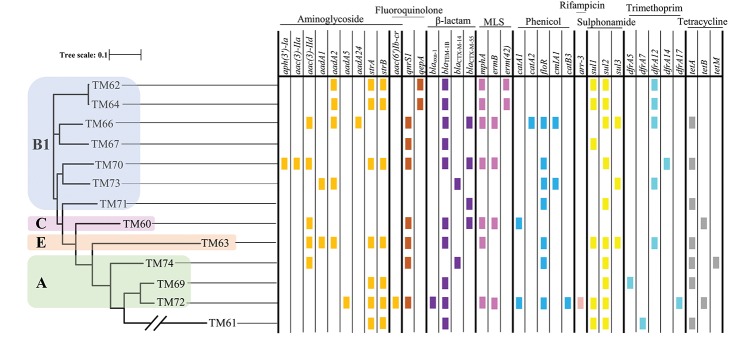
Phylogenetic relationship and distribution of different resistance genes among MCRPEn isolates. Based on single nucleotide polymorphisms, the 13 *E. coli* MCRPEn isolates were classified into four different phylogroups (A, B1, C, and E) as represented by differently colored boxes over the phylogenetic tree. The presence of different resistance genes that confer resistance toward each of the antimicrobials determined by WGS are shown as small colored rectangles. The two diagonal bars across a branch of the phylogenetic tree represent scale break. MLS, macrolide, lincosamide, and streptogramin B.

From the PFGE results, the CPE isolates were found to be clonally related; there are several previous reports describing their nosocomial transmission (Nordmann et al., 2011; Mataseje et al., 2016). However, all the MCRPEn isolates were clonally unrelated to each other as shown by PFGE; a high rate of MCRPEn carriage (5.9%) was also determined in this surveillance study. For a detailed understanding of the genomic characteristics of MCRPEn isolates, whole genome sequencing (WGS) was carried out. WGS of MCRPEn isolates showed that all MCRPEn isolates belonged to different individual sequence types except two *E. coli* isolates (TM62 and TM64) both of which belong to ST424 (Table 2). The phylogenetic analysis based on single nucleotide polymorphisms showed that the *E. coli* isolates (*n* = 13) of the MCRPEn are diverse and belong to four different phylotypes—A, B1, C, and E (Figure 3). One isolate (TM61) does not belong to any of the phylotypes. The phylogenetic analysis also showed that TM62 and TM64 were more closely related than any other MCRPEn isolates. In addition to the *mcr-1* gene, the MCRPEn isolates were found to possess several resistance genes for a diverse range of antibiotics (Figure 3). S1 nuclease-PFGE followed by Southern hybridization showed that two *E. coli* MCRPEn isolates (TM63 and TM66) carried the *mcr-1* gene in their chromosome. All other 13 MCRPEn possess the *mcr-1* gene in the plasmids of two different sizes: ~33 and ~60 kb (Figure S1).

**Table 2:** Description of *mcr-1* gene location, plasmid analysis, and other characteristics of the MCRPEn isolates.

**Isolate id**	**Species**	**Sequence type**	**MIC of CST (μg/ml)**	**Location of *mcr-1***	**Plasmid replicon**	**Plasmid size (kb)**	**Query coverage (Identity) (%)**	**Reference accession no**.
TM60	*E. coli*	ST410	4	Plasmid	IncX4	~33	98.3 (100)	CP02404.1
TM61		ST131	16	Plasmid	IncX4	~33	99.1 (100)	CP02404.1
TM62		ST424	16	Plasmid	IncI2	~68	90.3 (99.8)	CP025679.1
TM63		ST1011	16	Chromosome	NA	NA	NA	NA
TM64		ST424	8	Plasmid	IncI2	~68	89.9 (99.8)	CP025679.1
TM66		ST4014	8	Chromosome	NA	NA	NA	NA
TM67		ST156	8	Plasmid	IncI2	~60	97.2 (99.9)	MF978388.1
TM69		ST4741	4	Plasmid	IncI2	~60	98.2 (99.8)	MF978388.1
TM70		ST448	8	Plasmid	IncX4	~33	98.4 (100)	CP02404.1
TM71		ST1196	4	Plasmid	IncI2	~60	98.9 (99.9)	MF978388.1
TM72		ST744	8	Plasmid	IncX4	~33	98.9 (100)	CP02404.1
TM73		ST3695	8	Plasmid	ND	~60	NA	NA
TM74		ST8335	8	Plasmid	IncX4	~33	98.9 (100)	CP02404.1
TM65	*K. pneumoniae*	ST394	16	Plasmid	IncI2	~68	90.4 (99.8)	CP025679.1
TM68		ST709	8	Plasmid	IncX4	~33	98.8 (99.9)	CP02404.1

*CST, colistin; NA, not applicable; ND, not determined*.

Table 2 shows that out of the 13 isolates carrying *mcr-1* in plasmids, two types of plasmids, IncX4 (*n* = 6) and IncI2 (*n* = 6) were found by WGS analysis. Along with *mcr-1* gene, both the IncX4 and IncI2 plasmids carried a wide range of the different variants of other resistance genes for β-lactams, fluoroquinolones, aminoglycosides, macrolides, phenicol, sulphonamides, tetracyclines etc. (Figure 3). One of the specimens from which two MCRPEn (TM64-*E. coli* and TM65-*K. pneumoniae*) were isolated carries IncI2 plasmids whereas the other specimen with the two isolates, TM67- *E. coli* and TM68- *K. pneumoniae* carries IncI2 and IncX4, respectively. The *mcr-1* genes in all six IncX4 plasmids (~33 kb) were found to possess a single point mutation (C27T) without causing any amino acid substitution. Interestingly, the same point mutation was observed in the previously reported plasmid carrying *mcr-1* in *K. pneumoniae* (accession no. CP024041.1) isolated from Thailand. This reference plasmid also has both a high query coverage and identity of 99% with the IncX4 plasmids identified in our study. Among the six IncI2 plasmids, three (TM62, TM64, TM65) were similar to the IncI2 plasmid (~68 kb) carrying *mcr-1* in an *Escherichia albertii* isolate from China (accession no. CP025679.1). Likewise, the other three IncI2 plasmids in TM67, TM69, and TM71 were similar to the *mcr-1*-carrying IncI2 plasmid (~60 kb) found in an *E. coli* isolate from China (accession no. MF978388.1). In one isolate (TM73), the contig with *mcr-1* (~11 kb) showed high similarity to the previously reported IncI2 plasmids and S1 nuclease-PFGE showed that *mcr-1* is located in a ~60 kb plasmid which is similar to the size of IncI2 plasmids identified in other MCRPEn isolates in our study. However, no IncI2 replicon was identified in TM63 by WGS and hence the type of plasmid carrying *mcr-1* could not be determined.

### Efficacy of PCR-Dipstick in Detecting MCRPEn and CPE as Evaluated During This Surveillance Study

Evaluation of the sensitivity and specificity of PCR-dipstick compared to conventional PCR, showed that PCR-dipstick had both 100% sensitivity and specificity in detecting the MCRPEn directly from the 219 rectal swab specimens examined in this study (Table 3). Likewise, it showed 100% sensitivity and 98.9% specificity in detecting CPE directly from clinical specimens (Table 3). The conventional method requires all the 219 specimens to be screened for the presence of MCRPEn and CPE isolates followed by confirmation of the presence of resistance genes which takes ~3–4 days (Figure 1). In contrast, PCR-dipstick extensively reduced the number of specimens which needed to be subjected for the isolation of MCRPEn and CPE from 219 to 34. Furthermore, PCR-dipstick was found to be rapid in detecting the presence of resistance genes thereby identifying the MCRPEn- and CPE-positive specimens in <2 h.

**Table 3:** Sensitivity and specificity of PCR-dipstick vs. conventional PCR for 219 rectal swab specimens.

		**Conventional PCR**		**Sensitivity (95% CI)**	**Specificity (95% CI)**
		**Positive**	**Negative**		
PCR-dipstick (MCRPEn)	Positive	13	0	100 (75.3–100)	100 (98.2–100)
	Negative	0	206		
PCR-dipstick (CPE)	Positive	19	2	100 (82.4–100)	98.9 (96.4–99.9)
	Negative	0	198		

*CI, confidence interval*.

### Risk Factors Associated With MCRPEn and CPE Carriage

The univariate analysis showed that chronic kidney disease (*p* = 0.02) alone was significantly associated with MCRPEn carriage (Table 4). On the other hand, diaper usage (*p* = 0.02), health complications such as chronic respiratory diseases (*p* = 0.009) and chronic kidney diseases (*p* = 0.02), invasive procedures of mechanical ventilation (p = 0.02), and tracheostomy (*p* = 0.003), history of multidrug-resistant organisms (*p* = 0.002) and prior exposure to antimicrobials (*p* = 0.05) were significantly associated with CPE carriage (Table 4).

**Table 4:** Risk factors associated with MCRPEn and CPE carriage.

**Characteristics**	**MCRPEn**		**CPE**	
	**OR (95% CI)**	***p*-value**	**OR (95% CI)**	***p*-value**
Diaper use	0.61 (0.16–2.28)	0.56	3.21 (1.23–8.38)	0.02
**Health complications**				
Chronic respiratory diseases	0.60 (0.08–4.84)	1.00	4.15 (1.42–12.13)	0.009
Chronic kidney diseases	3.95 (1.26–12.32)	0.02	3.10 (1.19–8.09)	0.02
**Invasive procedures**				
Mechanical ventilation	1.84 (0.54–6.30)	0.30	3.31 (1.24–8.82)	0.02
Tracheostomy	0.73 (0.10–5.93)	1.00	5.31 (1.78–15.85)	0.003
Carriage of MDROs^a^ (<3 months)	1.28 (0.38–4.34)	0.75	4.60 (1.75–12.12)	0.002
Exposure to antimicrobials^b^ (<3 months)	2.29 (0.29-18.21)	0.70	^**^	0.05

*OR, odds ratio; CI, confidence of interval; MDROs, multidrug-resistant organisms*.

a *MDROs include methicillin-resistant Staphylococcus aureus, vancomycin-resistant Enterococci, multidrug-resistant Pseudomonas aeruginosa, multidrug-resistant Acinetobacter baumannii, and extended-spectrum β-lactamase producers*.

b *Antimicrobials include β-lactams, β-lactamase inhibitors, quinolones, tetracyclines, glycopeptides, colistin, sulfamethoxazole-trimethoprim, clindamycin, and metronidazole*.

** *All 19 cases of CPE were exposed to antimicrobials*.

## Discussion

Due to their resistance toward the drugs of last resort, colistin/carbapenem, *mcr-1*, and carbapenemase co-producing *Enterobacteriaceae* have been reported from different parts of the world (Pulss et al., 2017; Mendes et al., 2018). Since there are no alternate therapeutics available, their rapid spreading has become an alarming threat which needs to be controlled. It is essential to understand the prevalence of both in healthcare settings to prevent their transmission. Recently, Jin et al. reported the simultaneous screening of MCRPEn and CPE from sewage water in China (Jin et al., 2018). However, there were no reports of the screening of MCRPEn together with CPE in hospitalized patients. We conducted a surveillance of MCRPEn and CPE together using the direct clinical specimens and divulged the MCRPEn (5.9%) and CPE (8.7%) carriage rate in a tertiary care hospital in Thailand by applying the PCR-dipstick-oriented specimen-direct surveillance strategy. Moreover, we demonstrated that the underlying medical conditions of MCRPEn and CPE carriage differ in that hospital.

CPE isolated in this study were found to carry only two types of the six carbapenemase genes tested—*bla*_NDM−1_, *bla*_OXA−232_, or both and they showed high level of resistance to most of the antibiotics tested. From the PFGE pattern, the clonal relatedness of the CPE isolates suggested that they have been spreading in the hospital within and between the wards. Thus, it becomes obvious that there is nosocomial transmission of CPE. These results are in line with the previous reports on the nosocomial transmission of CPE in clinical settings (Onori et al., 2015; Sotgiu et al., 2018). Furthermore, the currently determined high carriage rate (8.7%) of CPE is suspected to increase in the next few years if left uncontrolled.

Regarding MCRPEn, all of the MCRPEn (100%) isolated in this study were found to be colistin-resistant. MCRPEn identified in this study were all unrelated clones, except two *E. coli* isolates, which indicates that the MCRPEn isolates were acquired from outside the hospital. MCRPEn isolates could also have been acquired from other wards of the hospital which were not included in this surveillance study. WGS showed that the MCRPEn isolates carry two types of *mcr-1*-carrying plasmids, IncX4, and IncI2, which are also known to be two of the major plasmid types that mediate the spread of *mcr-1* gene (Cui et al., 2017; Li et al., 2017). The high identity of IncX4 plasmids with the previously reported IncX4 plasmid along with the same single point mutation detected in the *mcr-1* gene, suggests that IncX4 carrying *mcr-1* might be spreading in Thailand, thereby mediating MCRPEn dissemination. Similarly, the *mcr-1*-carrying IncI2 plasmids were highly identical to the previously reported IncI2 plasmids identified from food sources in China. Thus, the *mcr-1*-bearing IncI2 plasmids might have been imported from China; however, this association between the plasmids needs to be further elucidated. On the other hand, plasmid mediated spread of *mcr-1* gene between isolates would also have occurred in the intestinal flora of the patients and/or the isolates residing in the hospital environment. Furthermore, ST1011 and ST4014 *E. coli* isolates carrying *mcr-1* in the chromosome were epidemiologically unrelated to the previously reported *mcr-1*-positive *E. coli* isolates with the same sequence types (Guenther et al., 2017; Wang et al., 2017a). Altogether, these results suggest that MCRPEn would have been acquired from the community.

The identification of single specimens carrying two MCRPEn isolates with same or different types of plasmids shows the plausibility of the transfer of *mcr-1* gene from MCRPEn to naïve *Enterobacteriaceae* (Wang et al., 2018). In addition, the presence of both MCRPEn and CPE in the same specimen suggests that there is a risk of plasmid-mediated transfer of the *mcr-1* gene from MCRPEn to CPE or carbapenemase genes from CPE to MCRPEn, thereby leading to the emergence of carbapenem- and colistin-resistant *Enterobacteriaceae* without any therapeutic options (Beyrouthy et al., 2017; Pulss et al., 2017; Mendes et al., 2018). We showed a very high rate of MCRPEn (5.9%) even without any duplicated specimens whereas other studies showed a low rate of clinical MCRPEn (Yu et al., 2016; Chan et al., 2018). This might be due to the high sensitivity of PCR-dipstick in detecting the MCRPEn directly from clinical specimens, whereas the other methods, such as screening with selective media, are less sensitive.

Our risk factor analysis shows that only the chronic kidney disease is associated with the carriage of MCRPEn in this hospital. In contrast, the patients colonized with CPE have health complications such as chronic respiratory and kidney diseases, used diaper frequently, underwent invasive procedures and had prior history of infection with multidrug-resistant organisms and antibiotics exposure. These backgrounds in patients with CPE carriage are highly consistent with the previous reports (Han et al., 2017; McConville et al., 2017; Yamamoto et al., 2017a).

The main advantage of using PCR-dipstick is its rapidity to detect the presence of these multidrug-resistant pathogens by targeting the resistance genes directly from the clinical specimens within 2 h. We compared PCR-dipstick-oriented surveillance with the conventional method for the surveillance of MCRPEn and CPE and found that the conventional method is more time-consuming and laborious than PCR-dipstick-oriented surveillance. For instance, the rectal swab specimens examined in this study showed the growth of a mixture of several Gram-negatives including *mcr-1*-negative isolates in the selective media leading to the screening of all the suspected isolates. However, the specimen-direct detection of resistance genes by PCR-dipstick aids in the culturing of only the specimens that were found to be positive for the resistance genes thereby reducing the number of specimens for screening of MCRPEn and CPE (Figure 1). Since PCR-dipstick could simultaneously identify MCRPEn and CPE from direct clinical specimens by targeting the resistance genes within 2 h, it could aid in the faster implementation of infection control measures such as patient cohorting to prevent their dissemination.

The limitation of applying PCR-dipstick-oriented surveillance is that the *Enterobacteriaceae* that exhibit carbapenem and colistin resistance through mechanisms other than carbapenemase and *mcr-1* genes could not be detected unlike the detection systems which target the genes that mediate other modes of resistance. However, the risk of dissemination of carbapenem and colistin resistance resides in the plasmid-mediated transfer of carbapenemase and *mcr-1* genes, respectively (van Duin and Doi, 2017; MacNair et al., 2018). Considering this aspect of MCRPEn and CPE, the currently proposed and examined surveillance strategy will be indispensable for both the clinical and environmental screening of these superbugs. As it is feasible to design the PCR-dipstick technique for any other newly reported variants of carbapenemase and *mcr* genes by designing the primers and evaluating the optimal hybridization conditions, CPE and MCRPEn harboring other resistance genes could be detected with a customized dipstick (Shanmugakani et al., 2017). Two specimens that were identified as *bla*_IMP_-positive by PCR-dipstick did not show the growth of any CPE isolates on the selective media which might be due the *bla*_IMP_ carried by non-fermenters such as *Acinetobacter baumannii, Pseudomonas aeruginosa*, for example. Due to fewer number of MCRPEn- and CPE-positive cases, we could not apply a multivariate approach for the statistical analysis. Thus, a multiplicity could not be adjusted appropriately, influencing the interpretation of putative statistically significant findings. To determine the risk factors for MCRPEn and CPE in a hospital setting, a prospective, large-scaled study is warranted.

In summary, the MCRPEn isolates are undetectably existing in the hospital community due to the lack of a proper detection system. Our PCR-dipstick-oriented surveillance strategy is highly efficient in detecting MCRPEn directly from clinical specimens with a short turnaround time. The shockingly high prevalence of MCRPEn and CPE in the Thai hospital as determined in our surveillance study will be very helpful in undertaking potent infection control measures to control these deadly pathogens. Furthermore, a simple and cost-effective PCR-dipstick-oriented specimen-direct surveillance strategy for MCRPEn and CPE was developed and examined for its utility in clinical settings.

## Materials and Methods

### Ethics Statement and Specimen Collection

A total of 219 non-duplicated rectal swab specimens were collected using eSwab (COPAN Italia S.p.A., Brescia, Italy) from hospitalized patients (121 male and 98 female) with a mean age of 61 years in the surgical, medical, intensive care, intermediate care, cardiac care, and geriatric wards in Ramathibodi Hospital, Mahidol University in Bangkok, Thailand, during Jan–Feb 2018 as part of the Infection Control Committee cross-sectional surveillance activity. The rectal swabs collected were transported to the research laboratory and processed on the same day. Ethical approval was obtained from the Ethics Committee of Osaka University Graduate School of Medicine, Osaka, Japan and Ethical Clearance Committee on Human Rights Related to Research Involving Human Subjects, Faculty of Medicine Ramathibodi Hospital, Mahidol University, Bangkok, Thailand. Since rectal swab specimens were collected as a component of antimicrobial resistance surveillance in the hospital, the need for informed consent was waived by the Institutional Review Boards.

### Surveillance of MCRPEn and CPE Using PCR-Dipstick

PCR-dipstick was used for the direct detection of six carbapenemase genes (*bla*_NDM_, *bla*_KPC_, *bla*_IMP_, *bla*_OXA−48_, *bla*_VIM_, *bla*_GES_) and the *mcr-1* gene from the rectal swab specimens. We previously reported the development of this technique for the detection of four carbapenemase genes—*bla*_NDM_, *bla*_KPC_, *bla*_IMP_, and *bla*_OXA−48_, from direct clinical specimens (Shanmugakani et al., 2017). Here, we constructed another dipstick that could detect two additional carbapenemase genes, *bla*_VIM_, *bla*_GES_, and the *mcr-1* gene (Supplementary Material). Thus, two dipsticks were used for the detection of MCRPEn and CPE directly from rectal swab specimens (Supplementary Material and Figure S2).

For the isolation of MCRPEn and CPE isolates from the PCR-dipstick-positive specimens, an aliquot of 100 μl each of the rectal swab specimens was inoculated onto SuperPolymyxin and M-ECC media, respectively (Nordmann et al., 2016; Yamamoto et al., 2017b). The plates were then incubated at 37°C for 24~48 h and the suspected colonies were subjected to species identification and antimicrobial susceptibility testing for carbapenems and other β-lactams, fluoroquinolones, tetracyclines, aminoglycosides, and sulphonamides using MicroScan WalkAway 96 Plus (Beckman Coulter, CA, USA) with a Neg Combo NF1J panel. The resistance profile of the isolates were then characterized according to the guideline M100, 28th edition of Clinical and Laboratory Standards Institute (CLSI) (Wayne, PA, USA). For colistin, antibiotic susceptibility testing was performed by broth microdilution using cation-adjusted Mueller Hinton II Broth according to CLSI document M07-A10, 2015 and the MIC breakpoints (≤2 susceptible, >2 resistant) given by European Committee on Antimicrobial Susceptibility Testing (http://www.eucast.org/fileadmin/src/media/PDFs/EUCAST_files/Breakpoint_tables/v_8.1_Breakpoint_Tables.pdf) was followed for result interpretation (Chew et al., 2017; Simar et al., 2017). The presence of carbapenemase/*mcr-1* genes in the MCRPEn and CPE isolates was confirmed by conventional PCR and sequencing (Supplementary Material and Table S2). To analyze whether the MCRPEn and CPE isolates were clonally spreading in the hospital, they were subjected to PFGE using a previously reported protocol with slight modifications (Supplementary Material) (Ohno et al., 2017).

### Genomic Characterization of MCRPEn Isolates

To determine the location of the *mcr-1* gene in the MCRPEn isolates, S1 nuclease-PFGE followed by Southern hybridization with *mcr-1* probe was performed (Supplementary Material). For WGS, all the MCRPEn isolates were grown overnight at 37°C in brain heart infusion broth (BD Bacto, Franklin Lakes, NJ, USA) and their genomic DNA was isolated using DNeasy Powersoil Kit (QIAGEN, Valencia, CA, USA). From the isolated genomic DNA, the libraries were prepared with MiSeq v2 500 cycle kit (Illumina, San Diego, CA, USA) and subjected to the MiSeq system (Illumina) (Miyamoto et al., 2014). *De novo* assembly of the sequence reads was performed using CLC Genomics Workbench 11.0.1 (CLC Bio, Aarhus, Denmark). The assembled sequences were submitted to MLST 2.0, PlasmidFinder 1.3, and ResFinder 2.1 to determine the sequence types, plasmid replicons and presence of different resistance genes, respectively (Larsen et al., 2012; Zankari et al., 2012; Carattoli et al., 2014). The *mcr-1*-carrying contigs were extracted and searched for similar sequences using BLAST in the NCBI database (https://blast.ncbi.nlm.nih.gov/Blast.cgi). Then, the sequence identified with the highest query coverage/identity was taken as the reference sequence for further analysis. The contigs were mapped with the reference sequence on CLC Genomics Workbench and the resultant mapped sequences were compared with the reference sequence to determine their similarity. With the *E. coli* str. K-12 substr. MG1655 (accession no: NC_00913.3) as the reference genome, phylogenetic relationships among the MCRPEn (*E. coli*) isolates were determined based on single nucleotide polymorphisms using CSI Phylogeny 1.3 (Kaas et al., 2014). Their phylotypes were determined based on the previously described phylo-typing method (Clermont et al., 2013).

### Sequence Accession Numbers

The whole genome sequences of the 15 MCRPEn isolates identified in this study had been deposited to the DDBJ/ENA/GenBank under the accession numbers: DRX131210 (TM60), DRX131211 (TM61), DRX131212 (TM62), DRX131213 (TM63), DRX131214 (TM64), DRX131215 (TM65), DRX131216 (TM66), DRX131217 (TM67), DRX131218 (TM68), DRX131219 (TM69), DRX131220 (TM70), DRX131221 (TM71), DRX131222 (TM72), DRX131223 (TM73), and DRX131224 (TM74).

### Evaluation of the Efficacy of PCR-Dipstick-Oriented Surveillance

During the PCR-dipstick-oriented surveillance of MCRPEn and CPE, conventional method of surveillance was carried out simultaneously (Figure 1). For the conventional method, initially all the rectal swab specimens were inoculated into SuperPolymyxin and M-ECC media. Then, the suspected MCRPEn and CPE isolates were isolated and subjected to genomic DNA isolation followed by conventional PCR for carbapenemase and *mcr-1* genes (Supplementary Material). The efficiency of PCR-dipstick in detecting the MCRPEn and CPE directly in rectal swab specimens was determined by comparing the results of PCR-dipstick with a reference comparator in which the conventional PCR was performed for the genomic DNA of MCRPEn and CPE isolated from rectal swab specimens (Supplementary Material). The sensitivity and specificity of PCR-dipstick was calculated using GraphPad Prism version 6.05 (GraphPad Software, La Jolla, CA, USA). To evaluate the utility of PCR-dipstick-oriented surveillance, the work flow of PCR-dipstick-oriented method was compared with the conventional surveillance method (Figure 1).

### Risk Factor Analysis

Clinical characteristics of the patients were analyzed for the risk factors associated with MCRPEn and CPE carriage separately. Clinical characteristics such as age, sex, length of hospitalization, invasive procedures, history of hospitalization (< 90 days), and prior antibiotics exposure (<90 days) were included for risk factor analysis. Logistic regression, Chi-square test, Fisher's exact test were performed, as appropriate, to assess the carriage rate of MCRPEn and CPE with each factor. The variables with two-tailed *p* ≤ 0.05 were considered statistically significant. The statistical analysis of the risk factors was performed with StataMP 15 (Stata Corp., College Station, TX, USA).

## Author Contributions

YA and KT conceived and designed the study. RS, WL, NC, SS, NY, IN, HY, DT, NS, and PS contributed to the specimen collection, isolation, and the phenotypic/genotypic characterization of clinical isolates. RS and YS contributed to the whole genome sequencing. RS, HH, DM, and YF performed the statistical analysis. RS, YA, and KT drafted the manuscript drafting. YA, KM, PS, KT, and SH performed the critical revision of the manuscript for intellectual content. All the authors read, edited, and approved the final manuscript.

### Conflict of Interest Statement

The authors declare that the research was conducted in the absence of any commercial or financial relationships that could be construed as a potential conflict of interest.
