# Supplementary material for: PCR-Dipstick-Oriented Surveillance and Characterization of mcr-1- and Carbapenemase-Carrying Enterobacteriaceae in a Thai Hospital
Source: Front Microbiol. 2019 Feb 8;10:149. doi: 10.3389/fmicb.2019.00149 (PMC6375898; doi:10.3389/fmicb.2019.00149)
Supplement: Supplementary file 1 [file Table_1.docx]

***Supplementary Material***

**PCR-Dipstick-Oriented Surveillance and Characterization of *mcr-1*- and Carbapenemase-Carrying *Enterobacteriaceae* in a Thai Hospital**

**Rathina Kumar Shanmugakani, Yukihiro Akeda*, Yo Sugawara, Warawut Laolerd, Narong Chaihongsa, Suntariya Sirichot, Norihisa Yamamoto, Hideharu Hagiya, Daiichi Morii, Yoshihiro Fujiya, Isao Nishi, Hisao Yoshida, Dan Takeuchi, Noriko Sakamoto, Kumthorn Malathum, Pitak Santanirand, Kazunori Tomono* and Shigeyuki Hamada**

*** Correspondence:**

Dr. Yukihiro Akeda: akeda@biken.osaka-u.ac.jp

Dr. Kazunori Tomono: tomono@hp-infect.med.osaka-u.ac.jp

**SUPPLEMENTARY MATERIALS AND METHODS**

**Construction of PCR-Dipstick for *bla*_VIM_, *bla*_GES_ and *mcr-1***

The establishment of PCR-dipstick began with primer designing for the three target genes, namely, *bla*_VIM_, *bla*_GES_ and *mcr-1*, using reference sequences with the accession numbers AY152821, AF156486, and KX458104, respectively. The primers were designed to detect the different variants of each gene. After examining the different primer sets of each gene for multiplex compatibility, the optimal primer sets were chosen. Then, either the forward or reverse primer of each primer set was labelled with a distinct tag-linker sequence (T2/T3/T4) and biotin (B) (Tohoku Bio-Array, Sendai, Japan). The labelled primers are as follows: *bla*_VIM_ primers, VIM-F (5’-T4-GTTTGGTCGCATATCGCAAC-3’) and VIM-R (5’-B-AATGCGCAGCACCAGGATAG-3’) for *bla*_VIM-1-4,6-15,17-20_; *bla*_GES_ primers, GES-F (5’-B-GCTTCATTCACGCACTATT-3’) and GES-R (5’-T3-CGATGCTAGAAACCGCTC-3’) for *bla*_GES-1-9,11-20_; *mcr-1* primers, MCR-1-F (5’-B-ACTTATGGCACGGTCTATGA-3’) and MCR-R (5’-T2-ATACTGGCAAGCTTACCCAC-3’) for *mcr-1*,*­-1.2*,*-2*.

The PCR-dipstick for *bla*_VIM_, *bla*_GES_ and *mcr-1* was examined with the positive control strains, VIM-1-producing *Klebsiella pneumoniae* NCTC 13440, VIM-2-producing *Pseudomonas aeruginosa* from Japan, GES-5-producing *P. aeruginosa* from Japan and MCR-1-producing *Escherichia coli* from Thailand using the previously described protocol (Shanmugakani et al., 2017). After the validation of PCR-dipstick with the positive control genomic DNA and bacterial cultures, it was studied for its utility for direct clinical specimens using spiked stool specimens. The results of PCR-dipstick for the spiked stool specimens suggested its use for direct clinical specimens. Thus, the PCR-dipstick for the direct detection of *bla*_VIM_, *bla*_GES_, and *mcr-1* from clinical specimens was constructed.

**PCR-Dipstick for Detection of Resistance Genes from Rectal Swab Specimens**

An aliquot of 100 μl of the rectal swab specimens was boiled for 5 min. and centrifuged at 13,000 rpm for 5 min. Then, 5 μl of the supernatant was used as the template for multiplex PCR with the labelled primers for *bla*_NDM_, *bla*_KPC_, *bla*_IMP_, and *bla*_OXA-48_ (Shanmugakani et al., 2017). Simultaneously, another 5 μl of the supernatant was taken for the multiplex PCR for *bla*_VIM_, *bla*_GES_ and *mcr-1* genes. The PCR products of the two multiplex PCR were subjected to dipstick development individually, as described previously (Shanmugakani et al., 2017). Thus, two dipsticks - dipstick 1 (*bla*_NDM_, *bla*_KPC_, *bla*_IMP_, and *bla*_OXA-48_) and dipstick 2 (*bla*_VIM_, *bla*_GES_, and *mcr-1*) were developed to detect the presence of six carbapenemase and *mcr-1* genes directly from rectal swab specimens within two hours.

**Characterization of Carbapenemase and *mcr-1* Genes in Clinical Isolates**

For the molecular characterization of carbapenemase and *mcr-1* genes, the MCRPEn and CPE isolates were inoculated into Luria-Bertani broth (Sigma-Aldrich, St. Louis, MO, USA) and incubated overnight at 37°C with shaking. Using QIAamp DNA Mini Kit (QIAGEN, Valencia, CA, USA), genomic DNA was isolated according to the manufacturer’s protocol. Conventional PCR was performed for the identification of six carbapenemase (*bla*_NDM_, *bla*_KPC_, *bla*_IMP_, *bla*_OXA-48_, *bla*_VIM_, *bla*_GES_) and *mcr-1* genes. The primers used for conventional PCR are listed in Supplementary Table S2 and the PCR conditions were the same as described previously (Shanmugakani et al., 2017). The results of conventional PCR were used as the reference comparator for the calculation of sensitivity/specificity of PCR-dipstick in detecting those resistance genes directly from rectal swab specimens. After confirming the presence of the carbapenemase and *mcr-1* genes in the clinical isolates, the full gene was amplified and subjected to Sanger sequencing for the identification of their allelic variants. Then, the sequencing results were compared with the sequences of their different allelic variants retrieved from GenBank (National Center for Biotechnology Information, National Institutes of health, Bethesda, MD) using GENETYX ver.11 (Genetyx Co., Tokyo, Japan).

**Pulsed-Field Gel Electrophoresis of MCRPEn and CPE**

Pulsed-field gel electrophoresis (PFGE) was performed for the clinical isolates with the procedures as described before with slight modifications (Ohno et al., 2017). The plugs containing bacterial cells were prepared using low melt agarose (Bio-Rad Laboratories, Inc., Tokyo, Japan) and lysed with 2 mg/ml of lysozyme (Wako Pure Chemical Industries, Osaka, Japan) for 4~6 hours at 37°C and treated with 1 mg/ml of proteinase K (Wako Pure Chemical Industries) overnight at 55°C. Then, the plugs were treated with benzylsulfonyl fluoride (Wako Pure Chemical Industries) for 1 hour at 55°C followed by 5~10 min. in ice and a restriction digestion was performed overnight using *Xba*I (Takara Bio Inc., Shiga, Japan) with 50 U/plug at 37°C. After each of the reaction steps, the plugs were washed well with TE (10 mM Tris-HCl, 1 mM EDTA, pH-8.0) buffer. Electrophoresis of the plugs was performed along with CHEF DNA size standard DNA Lambda ladder (Bio-Rad Laboratories, Inc.) in 1% SeaKem Gold agarose (Lonza, Rockland, ME, USA) at the same conditions with the parameters as described before using CHEF Mapper system (Bio-Rad Laboratories, Inc.) (Ohno et al., 2017). Following electrophoresis, the gel was washed with sterile water for 1 hour and stained with gel red solution for 1 hour. After staining, the gel was visualized under the UV transilluminator. The dendrogram was drawn using BioNumerics version 7.5 (Applied Maths, Austin, TX, USA) and the isolates with Dice similarity index ≥ 85% were considered to belong to the same cluster.

**S1 Nuclease-PFGE of MCRPEn**

Plug preparation was performed similar to that of PFGE with *Xba*I digestion as mentioned above, however, the treatment with *Xba*I was replaced with S1 nuclease. After treating the plugs with benzylsulfonyl fluoride, they were incubated in deionized water for 30 min. at room temperature. Then, the plugs were incubated with S1 nuclease (0.5 U/μl) in ice for 30 min. and 23°C for 30 min. After S1 nuclease treatment, the plugs were incubated with 0.5 M EDTA (pH-8.0) for 5 min. and washed with TE buffer. Finally, the plugs were incubated with 0.5× TBE (Tris/boric acid/EDTA) buffer (Bio-Rad Laboratories, Inc.) on ice for 1 h. Electrophoresis was performed at the same conditions mentioned above with a change in autoalgorithm at 20-250 kb range and run time for 15 h. Then, the gel was subjected to Southern hybridization using digoxigenin-labelled *mcr-1* probe according to the manufacturer’s instructions with slight modifications (Roche Diagnostics, GmbH, Germany).

**REFERENCES**

Shanmugakani, R. K., Akeda, Y., Yamamoto, N., Sakamoto, N., Hagiya, H., Yoshida, H., et al. (2017). Pcr-dipstick chromatography for differential detection of carbapenemase genes directly in stool specimens. *Antimicrob. Agents Chemother.* 61(6). doi: 10.1128/AAC.00067-17

Ohno, Y., Nakamura, A., Hashimoto, E., Matsutani, H., Abe, N., Fukuda, S., et al. (2017). Molecular epidemiology of carbapenemase-producing *Enterobacteriaceae* in a primary care hospital in Japan, 2010-2013. *J. Infect. Chemother.* 23(4)**,** 224-229. doi: 10.1016/j.jiac.2016.12.013

Monteiro, J., Widen, R. H., Pignatari, A. C., Kubasek, C. and Silbert, S. (2012). Rapid detection of carbapenemase genes by multiplex real-time PCR. *J. Antimicrob. Chemother.* 67, 906-909. doi: 10.1093/jac/dkr563

Hong, S. S., Kim, K., Huh, J. Y., Jung, B., Kang, M. S., and Hong, S. G. (2012). Multiplex pcr for rapid detection of genes encoding class a carbapenemases. *Ann. Lab. Med.* 32(5)**,** 359-361. doi: 10.3343/alm.2012.32.5.359

**Table S1│**Antimicrobial resistance profiles of MCRPEn and CPE isolated from the rectal swab specimens.

| **Antimicrobials** | **No. of resistant isolates (%)** | | | | | | |
| --- | --- | --- | --- | --- | --- | --- | --- |
|  | **MCRPEn (N=15)** | | |  | **CPE (N=19)** | | |
|  | ***E. coli* (n=13)** | ***K. pneumoniae***  **(n=2)** | **Total** |  | ***K. pneumoniae***  **(n=18)** | ***C. farmeri* (n=1)** | **Total** |
| TZP | 1 (7.7) | 0 | 1 (6.7) |  | 18 (100) | 1 (100) | 19 (100) |
| PIP | 13 (100) | 1 (50) | 14 (93.3) |  | 18 (100) | 1 (100) | 19 (100) |
| CAZ | 4 (30.8) | 1 (50) | 5 (33.3) |  | 18 (100) | 1 (100) | 19 (100) |
| FEP | 5 (38.5) | 1 (50) | 6 (40) |  | 18 (100) | 1 (100) | 19 (100) |
| ATM | 4 (30.8) | 1 (50) | 5 (33.3) |  | 18 (100) | 1 (100) | 19 (100) |
| IPM | 0 | 0 | 0 |  | 15 (83.3) | 1 (100) | 16 (84.2) |
| MEM | 0 | 0 | 0 |  | 17 (94.4) | 1 (100) | 18 (94.7) |
| DOR | 0 | 1 (50) | 1 (6.7) |  | 16 (88.9) | 1 (100) | 17 (89.5) |
| GEN | 5 (38.5) | 0 | 5 (33.3) |  | 6 (33.3) | 0 | 6 (31.6) |
| AMK | 0 | 0 | 0 |  | 7 (38.9) | 0 | 7 (36.8) |
| TOB | 6 (46.2) | 1 (50) | 7 (46.7) |  | 16 (88.9) | 1 (100) | 17 (89.5) |
| MIN | 3 (23.1) | 2 (100) | 5 (33.3) |  | 17(94.4) | 0 | 17 (89.5) |
| FOF | 2 (15.4) | 2 (100) | 4 (26.7) |  | 17 (94.4) | 0 | 17 (89.5) |
| SXT | 9 (69.2) | 1 (50) | 10 (66.7) |  | 12 (66.7) | 0 | 12 (63.2) |
| CIP | 10 (77) | 1 (50) | 11 (73.3) |  | 18 (100) | 0 | 18 (94.7) |
| LVX | 10 (77) | 1 (50) | 11 (73.3) |  | 18 (100) | 0 | 18 (94.7) |
| CST | 13 (100) | 2 (100) | 15 (100) |  | 3 (16.7) | 0 | 3 (15.8) |

TZP, piperacillin-tazobactam; PIP, piperacillin; CAZ, ceftazidime; FEP, cefepime; ATM, aztreonam; IPM, imipenem; MEM, meropenem; DOR, doripenem, GEN-gentamycin, AMK, amikacin; TOB, tobramycin; MIN, minocycline; FOF, fosfomycin; SXT, trimethoprim-sulfamethoxazole; CIP, ciprofloxacin; LVX, levofloxacin; CST, colistin.

**Table S2│**Primers used for conventional PCR.

| **Target genes** | **Primer name** | **Primer sequence (5’-3’)** | **Amplicon size (bp)** | **References** |
| --- | --- | --- | --- | --- |
| *bla*_NDM_ | NDM-F | CGCAACACAGCCTGACTTT | 126 | Shanmugakani et al., 2017 |
|  | NDM-R | TCGATCCCAACGGTGATAT |  |  |
|  |  |  |  |  |
| *bla*_KPC_ | KPC-F | GGCAGTCGGAGACAAAACC | 177 | Shanmugakani et al., 2017 |
|  | KPC-R | GGCAGTCGGAGACAAAACC |  |  |
|  |  |  |  |  |
| *bla*_IMP_ | IMP-F | ACCGCAGCAGAGTCTTTGCC | 587 | Shanmugakani et al., 2017 |
|  | IMP-R | ACAACCAGTTTTGCCTTACC |  |  |
|  |  |  |  |  |
| *bla*_OXA-48_ | OXA-48-F | GTGGCATCGATTATCGGAAT | 176 | Shanmugakani et al., 2017 |
|  | OXA-48-R | GATGCGGGTAAAAATGCTTG |  |  |
|  |  |  |  |  |
| *bla*_VIM_ | VIM-F | GTTTGGTCGCATATCGCAAC | 382 | Monteiro et al., 2012 |
|  | VIM-R | AATGCGCAGCACCAGGATAG |  |  |
|  |  |  |  |  |
| *bla*_GES_ | GES-F | GCTTCATTCACGCACTATT | 323 | Hong et al., 2012 |
|  | GES-R | CGATGCTAGAAACCGCTC |  |  |
|  |  |  |  |  |
| *mcr-1* | MCR-1-F | ACTTATGGCACGGTCTATGA | 332 | This study |
|  | MCR-1-R | ATACTGGCAAGCTTACCCACCGA |  |  |
