# Supplementary figures and images for: PCR-Dipstick-Oriented Surveillance and Characterization of mcr-1- and Carbapenemase-Carrying Enterobacteriaceae in a Thai Hospital
Source: Front Microbiol. 2019 Feb 8;10:149. doi: 10.3389/fmicb.2019.00149 (PMC6375898; doi:10.3389/fmicb.2019.00149)

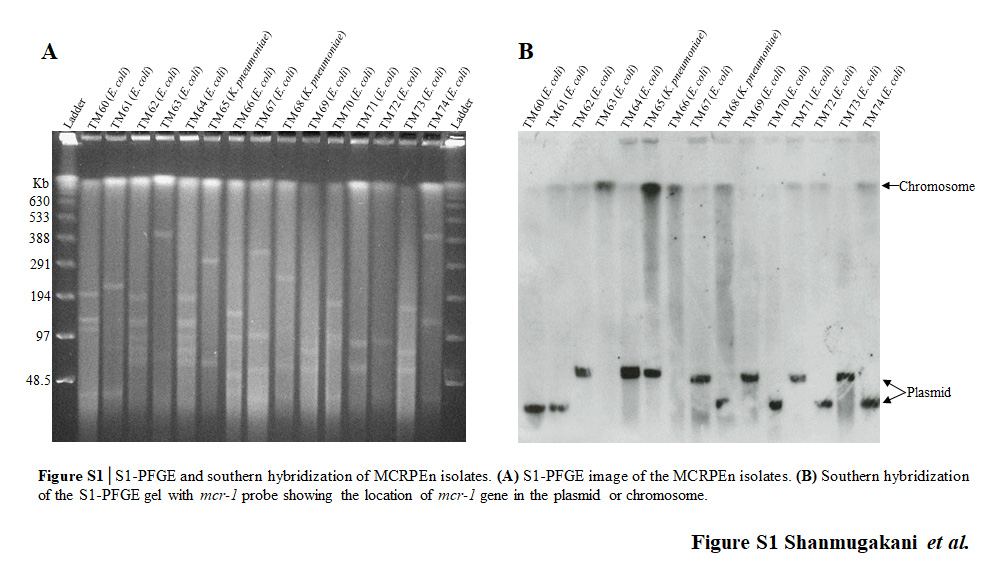

Supplement: Supplementary file 2 [file Image_1.JPEG]

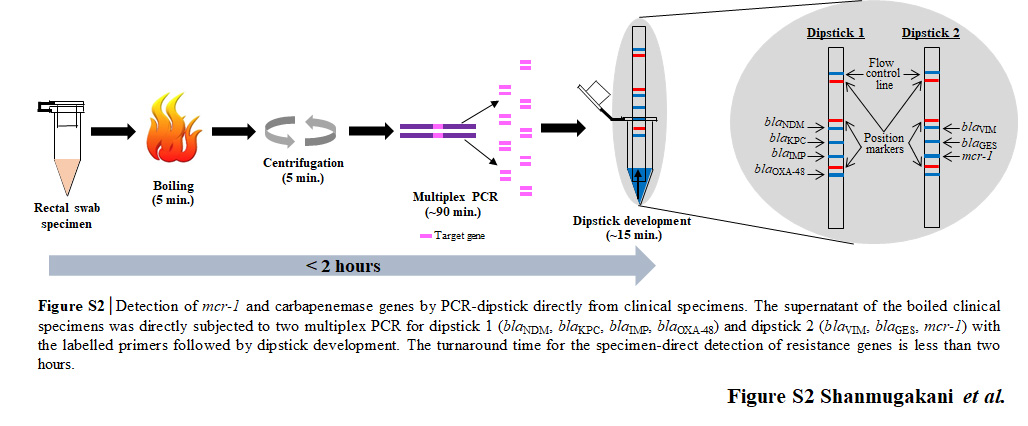

Supplement: Supplementary file 3 [file Image_2.JPEG]
